# A rare presentation of acute-onset chronic inflammatory demyelinating polyneuropathy with the detection of anti-GM3 and anti-sulfatides antibodies: a case report

**DOI:** 10.3389/fimmu.2024.1409637

**Published:** 2024-07-15

**Authors:** Ruohan Sun, Yao Meng, Lingyu Li, Wei-hong Chen, Jing Xu, Peiyuan Lv, Yanhong Dong

**Affiliations:** ^1^ Department of Neurology, Hebei General Hospital, Shijiazhuang, China; ^2^ Department of Neurology, Hebei Medical University, Shijiazhuang, Hebei, China; ^3^ Department of Neurology, Hebei North University, Zhangjiakou, Hebei, China; ^4^ Affiliated Hospital of North China University of Science and Technology, Tangshan, Hebei, China; ^5^ Department of Neurology, Hebei Provincial Key Laboratory of Cerebral Networks and Cognitive Disorders, Shijiazhuang, Hebei, China

**Keywords:** Guillain-Barré syndrome, chronic inflammatory demyelinating polyneuropathy, antibody, diagnose, demyelinating disease

## Abstract

**Objectives:**

Chronic inflammatory demyelinating polyneuropathy (CIDP) is an acquired immune-mediated neuropathy defined by clinical progression for more than 2 months. 16-20% of CIDP patients may present with rapidly progressive weakness that resembles GBS, known as acute-onset CIDP (A-CIDP). However, it is challenging to distinguish from GBS-TRF because of their similar clinical symptom and features. In this case review, we report a patient with A-CIDP with the detection of anti-GM3 and anti-sulfatides antibodies, which rarely have been in A-CIDP and may account for her progressive and recurrent symptoms.

**Methods:**

We analyzed existing medical literature and described a clinical case of A-CIDP with antibodies positive.

**Results:**

We reported a 56-year-old female presented with bilateral lower extremity weakness and distal numbness. She experienced similar symptoms four times and responded well to the IVIg therapy. Lumbar puncture demonstrated albumin-cytologic dissociation and EDX examination revealed multiple peripheral nerve damage. After ruling out other demyelination diseases, a diagnosis of A-CIDP was made.

**Discussion:**

The antiganglioside and anti-sulfatide antibodies are involved in CIDP pathogenesis and can help to distinguish A-CIDP and other variants. To prevent secondary damage, it is important to monitor relapse and remission symptoms along the treatment line. A rare case of A-CIDP is discussed concerning the detection of anti-GM3 and anti-sulfatides antibodies, thus making a retrospective comparison of antibodies in some literature to understand A-CIDP better.

## Introduction

Chronic inflammatory demyelinating polyneuropathy (CIDP) is a chronic immune-mediated neuropathy characterized by the progressive demyelination of the peripheral nerve fibers. Acute-onset CIDP (A-CIDP) occurs in 16–20% of CIDP patients, with rapidly progressive weakness developing within 8 weeks (1). Guillain-Barré syndrome (GBS), a progressive immune-mediated polyradiculoneuropathy, is characterized by typically symmetrical limb weakness and hyporeflexia with sensory impairment. Approximately 8 to 16% of patients with GBS may worsen after the first improvement, named Guillain-Barré syndrome with treatment-related fluctuation (GBS-TRF) (2). Distinguishing between these variants in the early phase is vital because they require different treatments. Here, we describe a rare case testing positive for anti-sulfatides antibody IgM and the GM3 antibody IgM to explore the possible mechanism.

## Case presentation

**Figure 1:**
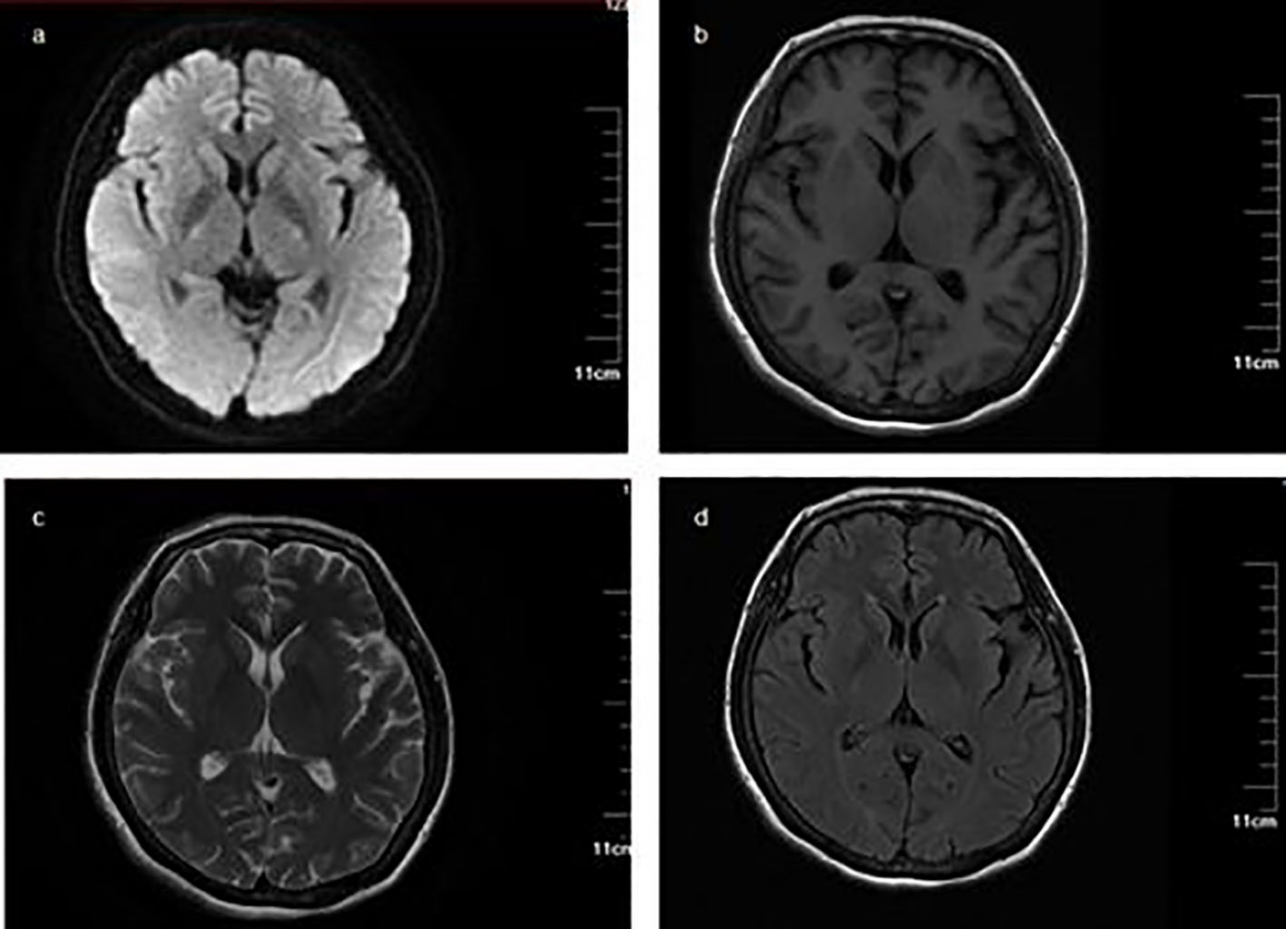
Magnetic resonance imaging and magnetic resonance angiography (MRA) MRI findings were unremarkable. **(A)** Diffusion-weighted imaging (DWI) sequence; **(B)** T1-weighted imaging; **(C)** T2-weighted imaging; **(D)** fluid-attenuated inversion recovery.

A 56-year-old female presented to the neurology department in our hospital with complaints of bilateral lower extremity weakness and distal numbness for 1 month that were gradually progressive and both hands developed numbness for 3 days. Before symptoms onset, she had flu-like symptoms 1 month previously. She was noted to have difficulty ambulating for a long distance with associated bilateral lower extremity soreness and distal paresthesia. On the sixth day, she recalled having a fever up to a maximum of 37.5°C without chilling; but associated with erythema on the trunk. She took oral antipyretic medication and the rash disappeared after 2 days. There were no other accompanying symptoms. She was normally alert and had lost approximately 10kg in 1 year. The right eye was in limited abduction inborn, without nystagmus or eye movement impediment. Sluggish to absent deep tendon reflexes were observed. The rest of the examinations were normal. The remainder of the routine laboratory tests and examinations were all within normal limits after admission except complement levels (complement C1q 149.8 mg/dL, 159–233 mg/dL). MRI findings were unremarkable (**Figure 1**). There were no signs of malignancy except myoma of the uterus. Electrodiagnosis (EDX) was done (**Tables 1**, **2**). Lumbar puncture demonstrated albumin-cytologic dissociation as evidenced by a cerebral spinal fluid (CSF) sample with normal cellularity and a protein level of 105.73 mg/dL (Normal range: 1–50 mg/dL). The pressure of CSF was 330mmH_2_O (Normal range: 80–180 mmH_2_O). Tests done for autoimmune antibodies showed GM3 IgM antibody in serum and the anti-sulfatides IgM antibody was found in CSF (Western blotting, Guangzhou Euroimmun Laboratory).

**Table 1:** Nerve conduction results.

	2/27/2023		3/14/2023		4/19/2023		6/7/2023		11/1/2023		12/1/2023	
	left	right	left	right	left	right	left	right	left	right	left	right
Median nerve												
Latency (ms)												
Wrist	4.3	5.4	4.1	4.5	4.5	5.5	4.1	4.6	5.23	6.49	5.3	5.4
Elbow	8.7	10	8.3	9.2	8.8	9.9	8.2	8.9	13	14.9	12.6	13.3
Amplitude (mV)												
Wrist	14.2	13.3	14.5	13.7	14.3	11.6	12.5	13.1	11.1	9	2.5	5.4
Elbow	13.4	12.6	13.6	13.6	11.4	10.3	10.4	12.7	9.3	8.2	2.4	3.7
Conduction velocity(m/s)												
Elbow	47.7	44.6	48.8	43.6	48.8	47.7	51.2	44.2	27.1	24.4	27.4	26.6
Distance(cm)												
Elbow	210	205	205	205	210	210	210	190	215	205	200	210
Peroneal nerve												
Latency (ms)												
Ankle	4.4	5.3	3.6	4.6	5.2	5.4	4.2	4.8	7.45	6.66	5.8	6
Fibula(head)	15.4	14.4	14	13.3	16.3	14.4	13.3	13.6	26.8	22.6	23.4	21.4
Amplitude (mV)												
Ankle	8.2	9.3	7.4	7.4	1.77	5.6	0.98	2.2	0.56	1.68	0.22	0.64
Fibula(head)	3.4	4.4	2.8	3.3	0.74	2.5	0.46	1.41	0.16	0.34	0.05	0.47
Conduction velocity(m/s)												
Fibula(head)	29.1	35.7	30.8	36.8	28.4	35.6	34.6	35.8	16.8	21.3	18.2	20.8
Distance(cm)												
Fibula(head)	320	325	320	320	315	320	315	315	325	340	320	320
Tibial nerve												
Latency (ms)												
Ankle	5.2	5.6	6	6.2	4.1	5.5	5.7	5.8	9.03	9.35	7.1	7.6
Knee	16	17	15.7	16	15.9	15.2	16.7	16.1	29.9	23.8	25.9	22.9
Amplitude (mV)												
Ankle	9	10.9	5.8	7	4.4	4.2	2.5	3.2	0.22	0.13	0.94	0.56
Knee	4.6	7.2	2.7	4.2	1.12	3	1.17	2.4	0.12	0.1	0.19	0.05
Conduction velocity(m/s)												
Knee	35.6	32.9	41.2	40.8	33.1	40.2	35.5	37.4	18.4	26.3	21	24.5
Distance(cm)												
Knee	385	375	400	400	390	390	390	385	385	380	395	375
M-Lat(ms)	7.3	10	6.6	6.5	6.9	4.9	7.7	6				
Mean F-M Lat	83	81.5	78	72.2	106	126	80.4	82.7				
Mean F-Lat	90.3	91.5	84.6	78.7	113	131	88.1	88				
Ulnar nerve												
Latency (ms)												
Wrist	3.9	3.3	3.7	2.9	2.8	3.6	3.8	3.3	5.23	4.83	3.9	4.2
Elbow	7.8	7.5	8.5	7.6	6.9	8	8.1	7.3	13	12.7	12.7	12.3
Amplitude (mV)												
Wrist	11	10.8	11.4	11.5	9.1	8.1	9.8	9.5	11.1	9.3	6.8	5.4
Elbow	10.5	9.3	10.7	10.6	8.8	8	8.7	7.9	9.2	7.5	3.1	4.8
Conduction velocity(m/s)												
Elbow	53.8	47.6	42.7	44.7	48.8	45.5	45.3	48.8	26.4	26.7	24.4	25.9
Distance(cm)												
Elbow	210	200	205	210	200	200	195	195	205	210	215	210

M-Lat, mean latency; Mean F-Lat, F-wave mean latency.

Reference range of normal motor nerve conduction velocity: median and ulnar nerve ≥50 m/s, tibial and peroneal nerve ≥ 40 m/s.

Reference range of normal latency: ≤3.5ms. Reference range of normal distal latency: median nerve ≤4ms, ulnar nerve ≤ 3ms, tibial nerve ≤ 6ms, peroneal nerve ≤ 5ms.

Reference range of normal distal amplitude: median nerve≥5mV, ulnar nerve≥5mV, tibial nerve≥5mV, peroneal nerve≥2.5mV.

Reference range of normal F-wave frequency of occurrence: except for the common peroneal nerve, it is generally 80%-100%, usually >50%.

EDX for the first time (2023–02-27) showed symmetrical peripheral nerve injury involving the upper and lower extremities with sensory and motor neuropathy with demyelinating features dominantly.

EDX for the second time (2023–04-19) showed an absence of sensory and motor neuropathy with demyelinating and axonal features dominantly.

EDX for the third time (2023–11-01) indicated multiple peripheral nerve damage in the extremities.

EDX for the fourth time (2023–12-01) revealed that the peripheral nerve polyradiculoneuropathy of extremities including axonal and demyelinating damage was more aggravated than before.

EDX (2023–03-14) and EDX (2023–06-07) were done in outpatient and revealed that the damage was alleviated.

**Table 2:** Sensory conduction nerve results.

	2/27/2023		3/14/2023		4/19/2023		6/7/2023	
	left	right	left	right	left	right	left	right
Median nerve								
Latency (ms)	3.8	NR	3.5	NR	NR	NR	3.5	4.3
Amplitude (mV)	5	NR	5	NR	NR	NR	1.7	1.07
Distance(cm)	140	–	145	–	–	–	145	145
Conduction velocity(m/s)	36.8	NR	41.4	NR	NR	NR	41.4	33.7
sural nerve								
Latency (ms)	2.4	2.5	2.6	2.1	1.78	1.93	NR	2.8
Amplitude (mV)	14.2	13.1	23.3	18.5	11.3	7.8	NR	3.1
Distance(cm)	105	5	125	100	95	95	–	110
Conduction velocity(m/s)	43.8	42	48.1	47.6	53.4	49.2	NR	39.3
ulnar nerve								
Latency (ms)	2.8	2.3	3	2.5	2.5	2.7	3	3.3
Amplitude (mV)	2.5	1.87	2.9	2.4	2.3	4.3	0.87	0.95
Distance(cm)	110	120	115	110	120	115	120	110
Conduction velocity(m/s)	39.3	52.2	38.3	44	48	42.6	40	33.3
superficial peroneal nerve								
Latency (ms)	1.85	2.2	2.1	2.1	NR	1.93	2.4	2.6
Amplitude (mV)	20.6	16.4	12.5	13.4	NR	3.6	7	8.7
Distance(cm)	95	105	95	95	–	95	100	110
Conduction velocity(m/s)	51.4	47.7	45.2	45.2	NR	49.2	41.7	42.3

This patient had no sensory examination at the third and fourth EDX. NR: not determined; –: nonexistent;

The reference range of normal sensory nerve amplitude: median nerve ≥ 7 μV, ulnar nerve ≥7 μV, sural nerve ≥ 7 μV.

The normal range of sensory nerve conduction velocity: median nerve ≥50 m/s, ulnar nerve ≥50 m/s, sural nerve ≥40 m/s.

The patient was initially diagnosed with acute inflammatory demyelinating polyneuropathy (AIDP). She received a standard single intravenous immunoglobulin (IVIg) dose (0.3 g/kg, 5 days). She responded well, which correlated with her symptoms alleviating quickly. She was discharged on day 15. She presented with aggravated bilateral lower extremity weakness 2 months later, demonstrated by being unable to complete a squat and falling down once, and distal extremity numbness like formication. Subsequently, she noted the onset of rapid numbness had progressed in both hands. She was readmitted to our hospital. Absent deep tendon reflexes were observed. The deep sensory function of the lower limbs was diminished. The groin below showed hyperalgesia. EDX was completed again (**Tables 1**, **2**). CSF showed a protein increase (145.74 mg/dL) with normal cell counts. The GM3 IgM antibody in serum was strongly positive and the anti-sulfatides IgM antibody had turned negative. She received a second IVIg therapy and corticosteroid therapy with methylprednisolone was initiated (80 mg/day for 3 days starting on hospital day 8), followed by an oral tablet (40mg/day). The methylprednisolone tablet dose was then gradually reduced, followed by 8 mg every week. She had intermittent symptoms of numbness in the feet only that did not interfere with daily life. When the dosage was adjusted to 4mg twice a day, similar symptoms reoccurred, worsening in the six months after her last discharge, and she was re-admitted to the hospital. CSF (albumin-cytologic dissociation) and EDX were done (**Tables 1**, **2**). Considering the diagnosis of A-CIDP, she received IVIG at 0.4/kg for 5 days followed by oral methylprednisolone tablets (40 mg/day) and mycophenolate mofetil tablets (0.5 g twice a day). Unfortunately, 2 weeks later, she presented with sudden lower limb weakness and numbness, predominantly aggravated on the left side, and EDX exacerbation (**Tables 1**, **2**). Ganglioside autoimmune antibodies had turned negative. There was limited improvement in numbness in the lower limbs after IVIg therapy. At a 3-month follow-up, she had normal strength with paresthesia improved and the symptoms had not fluctuated or reoccurred.

## Discussion

According to the latest clinical guidelines, typical CIDP is diagnosed as follows: developing over at least 8 weeks, symmetric muscle weakness and sensory involvement of limbs, and reduced tendon reflexes. Electrodiagnostic criteria include motor and sensory conduction abnormalities in at least two nerves (3). In addition to clinical and electrophysiological criteria, the disease is considered if the following conditions are fulfilled: albumin-cytologic dissociation and peripheral neuropathy excluding other causes, and most patients respond to immunotherapy except for demyelinating symmetric disease (DADS) with IgM M protein. Paraproteinemia is the presence of abnormal monoclonal globulin in the blood which can cause damage to sensory or sensorimotor nerves in DADS. Approximately 50% of patients have an antibody to myelin-associated glycoprotein (4). Peripheral neuropathy, which is predominantly sensory, non-neuralgia, demyelinating, and continuously progressing, should be highly suspected. There is significant variability in treatment and it fails to respond to intravenous immunoglobulin (IVIg) or corticosteroids. DADS without M protein are CIDP variants and are immunotherapeutic sensitive. Despite the elevation of cerebrospinal fluid protein in this patient, ESR and albumin were not elevated, and 24-hour urine protein quantification and serum immunoprotein electrophoresis were negative. Bone puncture and nerve biopsy were not performed because of the patient’s pain during diagnosis and treatment. Therefore, we were able to rule out the possibility of paraproteinemia. In typical CIDP, previous studies have shown symmetrical nerve root hypertrophy or increased signal intensity on T2-weighted MRI sequences (3) which indicates the low sensitivity and specificity of the diagnosis. Currently, neuro-ultrasound is useful in diagnosing chronic inflammatory neuropathies where electrophysiological criteria provide only a “possible” or “probable” diagnosis, such as in disease progression due to secondary axonal damage, or in patients with clinical suspicion of CIDP who may respond to treatment. Increased cross-sectional areas (CSAs), increased blood flow signals, echogenicity changes, and fasciculation enlargement were seen on high-resonance nerve ultrasound (HRUS) (5). There are quantitative assessments of nerve CSA including intranet and inner-nerve CSA variability and other parameters. Several scoring systems are used for the differentiation of CIDP such as the ultrasound pattern sum score (UPSS) and the Bochum ultrasound score (BUS). A UPSS of <3 had a negative predictive value of almost 100% for the exclusion of CIDP. The current study suggests that quantification of the extent of neuro enlargement using the utility of ultrasound pattern sub-score A (UPSA) is superior to CSA values alone in supporting CIDP, in particular a UPSA score of ≥7 (6, 7). In one study, a diagnostic pathway was developed to increase the specificity and sensitivity of the diagnosis of CIDP using a newly developed aBUS. If EFNS/PNS criteria are possible or probable and aBUS is at least 2, CIDP is suspected (7). As well as nerve cross-sectional area and distribution pattern, other ultrasound parameters such as nerve echogenicity play an important role and are prognostic. Even in short disease courses, increased echo intensity (class 2) appears to be a specific feature of CIDP (specificity 100%) (7). In CIDP, a hypoechogenic nerve is associated with edema and demyelination and clinical signs may remain stable or even improve with time, while a hyperechogenic nerve is associated with axonal damage with fibrous scarring and inflammatory infiltration and is more likely to show clinical deterioration. Furthermore, an ultrasound of the nerves may be of value in the management of patients with CIDP. Steroids may be more effective than IVIG in patients with normal or moderately enlarged CSA. Decreasing CSA following treatment may also indicate a better prognosis (8). Therefore, adding neuroimaging to nerve conduction study significantly improves the detection of CIDP with class IV evidence.

The initial neurological manifestations reached a nadir within 4 weeks, the reason being that she initially was diagnosed with GBS. Some notable findings in this patient include an initial presentation beyond 4 weeks, particularly three recurrent episodes, and the follow-up EDX studies which indicated that the final diagnosis was A-CIDP. However, it can be difficult to differentiate between a GBS patient experiencing a secondary deterioration after initial treatment and a patient with a second episode of weakness due to A-CIDP. Regrettably, the patient did not have sural nerve biopsies which may further support the diagnosis. Usually, a clinician is reliant on the timing of symptom onset and progression of the disease to differentiate these, for example, A-CIDP should be suspected when the symptoms relapse and remit or progress for more than 2 months or occur more than three times (9) and GBS-TRF has one or two post-treatment deteriorations, most within 9 weeks from onset, and has a more rapid onset of weakness. The median time to reach nadir and first and second exacerbation was significantly longer in the A-CIDP group compared to the GBS-TRF group (2). None of the patients with GBS-TRF deteriorated after 8 weeks. Ruts et al. compared 16 patients with GBS who had TRF and 8 patients with A-CIDP. The patients with A-CIDP were more likely to have sensory symptoms, manifested as worsening sensory ataxia, vibration, and pinprick sensation distributed in gloves and socks, and were less likely to have autonomic nervous system involvement, facial weakness, cranial nerve dysfunction disabled, or the need for mechanical ventilation. Furthermore, 50% of the A-CIDP patients could still walk independently during deteriorations (3). The patient in this case report was consistent with the results of the experiment. She presented paresthesia for limbs predominantly apart from cranial nerve involvement and autonomic nervous system involvement. She could walk independently although she presented with weakness in her lower limbs. It can be challenging to diagnose A-CIDP during the initial presentation. Currently, there is no prospective study that provides robust criteria to differentiate between recurrent GBS, GBS-TRF, and A-CIDP in the early phase of the disease.

CIDP is a demyelinating peripheral neuropathy, and neuro-electrophysiological detection is the most important adjunctive method. It mainly showed that the patient had multiple sensory and motor nerve damage in the limbs, and demyelination and axonal damage coexisted. The most important neurophysiological findings that gave rise to the suspicion of a demyelinating neuropathy are as follows: bilateral median, ulnar, common peroneal, and tibial nerves, with prolonged distal latency. A 50% reduction in the amplitude of the double common peroneal nerve suggested a proximal conduction block. The left tibial nerve showed reduced amplitude, and both tibial nerves displayed prolonged F-wave latency, indicating demyelination injury. The bilateral median and ulnar nerves showed decreased sensory nerve conduction velocity and/or amplitude. Demyelinating damage may be indicated by prolonged latency or reduced conduction velocity. The latency period, namely the terminal latency period of the distal muscle, is of great value in the diagnosis of demyelinating diseases. If the incubation period is longer than 3.5 ms, a conduction velocity that is slower than 70% of the normal value (i.e. upper limb <35 m/s; lower limb <28 m/s) and/or a distal latency that is greater than 130% of the normal value usually indicates myelin damage. Decreased amplitude of sensory potential does not differentiate between myelin and axonal damage: conduction velocity is slower than 70% of normal (i.e., upper limb <35 m/s; lower limb <28 m/s), and demyelinating damage may be suspected. Amplitudes are mainly axon alterations. The amplitude changes when the myelin sheath is damaged enough to uncover the axons. When the difference in amplitude between the distal end and the proximal end is half, a conduction block is considered to exist. The parameters of the F-wave of the upper extremity (median nerve) were the frequency of occurrence and the conduction velocity. The F-wave is useful for early diagnosis of peripheral neuropathy and to help localize the lesion. A decreased F-wave rate is the earliest manifestation of demyelinating disease. The EDX studies of the patient showed prominent demyelinating changes initially but gradually revealed axonal damage, probably due to continuous immune activation destroying axonal lesions. The relationship between demyelinating and axonal disorders is unclear but the symptoms are aggravated when axon damage exists. Also, complement C1q decreased in this patient which may be related to an immune imbalance in the body and result in damage to the cells.

The precise mechanisms of A-CIDP have been linked to activation of autoantibodies, T cells of the cellular immune response, complement proteins of the humoral immune response, and alteration of inflammatory cytokines (10). Autoimmune antibodies like gangliosides probably cause demyelinating polyneuropathy but the precise mechanism is unclear. LM1-containing ganglioside complexes are associated with specific clinical features reported in CIDP (11). It has been observed that CIDP patients have a higher incidence of IgM antibodies as compared to IgG antibodies. The most reported clinical reports are GM1, GD1a, GD1b, and GQ1b antibodies, while the GM3 antibody is less reported. A study by Meena A. Kannan et al. first discovered that antibody GM3 antibodies exist in children with AMAN and considered the presence of antiganglioside antibodies in serum not to be of much use in predicting the outcome (12). Chenghe Fan`s study revealed that GBS patients with GM3 IgM antibody had a significant association of facial nerve palsy, and sensory impairment (13). The GM3 IgM antibody detected in the patients was positive both times, and the clinical manifestations were mainly sensory disorders. Although there were no symptoms of facial paralysis, the study also suggested that patients with positive GM3 antibodies were more likely to have obvious sensory disorders, which was consistent with previous research findings. Many findings suggest that GM3 is involved in the development of cancer such as breast cancer and gastrointestinal tumors. The antibodies correlated with the paraneoplastic syndrome in CSF were not found. However, uterine myoma was found in the patient, which may be related to the GM3 antibody being positive in the body. Studies suggest that CIDP is caused by the production of autoantibodies against two or more peripheral nerve antigens rather than a single antigen. Different antibodies have different immunopathological mechanisms and clinical manifestations, resulting in different subtypes of CIDP. Laboratory testing for autoantibodies is now part of standard CIDP diagnosis and management, revealing multiple antibody reactivity using various testing methods. Whether the GM3 IgM antibody is associated with specific clinical features and pathogenetic mechanisms is not clear.

Sulfatide, a unique entity that differs from anti-ganglioside, can hold the nerve sheath membrane structure, regulate nerve impulses, and transmit membrane information. Anti-sulfatide antibody-related neuropathies present with multiple manifestations and differing neurophysiological characteristics which have been found in multiple sclerosis and Parkinson’s disease. The pathogenic mechanism of anti-sulfatide antibodies in peripheral neuropathy is still unclear, and high titer anti-sulfatide antibodies that expand the myelin space, combined with complement factor deposition, may be the cause of demyelinating changes in nerve axons. Common peripheral ganglioside antibodies are GM1, GM2, GM3, and GM4, accounting for 63.6% of all positive antibodies (14). There are not many clinical reports of anti-sulfatide antibodies because of the poor positive rate. Li Chen et al. reported a case characterized by cranial nerve involvement and anti-sulfatide IgM sulfatide antibodies, which was similar to our patient regarding the antibodies (15). Previous studies have shown that the positive rate of IgM anti-sulfatide antibodies in the cerebrospinal fluid of patients with CIDP was significantly different from that of the control group. Analysis of the correlation between CIDP patients’ serum and antibody levels in paired cerebrospinal fluid revealed no correlation. This suggests that anti-sulfatide antibodies in the serum and cerebrospinal fluid of GBS patients may have different sources and that the antibodies in the cerebrospinal fluid may originate in the sheath (16). Patients who are positive for anti-sulfatide antibodies are characterized by more severe distal symmetry damage, neurophysiological demyelination or axonal degeneration, a prolonged distal latency period, pain, and paresthesia as typical features. The patient was found to be positive for the anti-sulfatides antibody accompanied by being positive for anti-ganglioside antibodies. The coexistence of antibodies may predict delayed recovery when compared to the presence of antibodies alone. CSF protein probably reflects continuous anti-ganglioside antibody improvement. The anti-sulfatides IgM antibody in the patient was positive the first time and negative the second time, and the disease did not worsen during the follow-up of up to six months, suggesting that the transformation to being negative for the anti-sulfatides antibody can promote the recovery of the disease.

A study by Liselotte Rut et al. compared the patients with A-CIDP and GBS-TRF and concluded that patients with A-CIDP require long-term stable treatment with or without immunosuppressive agents to arrest inflammation and demyelination to prevent secondary axonal damage and patients with GBS-TRF require repeated IVIg courses that rely on drug-related pharmacokinetics to shorten the disease onset (2). IVIg treatment, which has the characteristics of rapid symptom improvement and a low risk of serious adverse events, was initially considered in this patient with recurrent exacerbations but individuals who exhibit indications of axonal damage may display resistance to treatment (1, 17). Studies have shown that high-dose oral dexamethasone shock therapy (4 days/month, 40mg/day) for 6 months is no better than conventional daily oral prednisolone (60mg/day, tapered over 8 months) (18). This suggests that the choice of a regimen with a lower cumulative dose may reduce the long-term adverse effects in patients, which is why we chose to use methylprednisolone at a conventional dose. Long-term use of glucocorticoids can lead to more serious side effects in CIDP patients with less disability. We did not use plasmapheresis because of the inconvenience and cost involved. Although there is still a lack of evidence-based guidelines for immunosuppressive therapy, some studies suggest that azathioprine, mycophenolate mofetil tablets, and cyclosporine may be selected as adjuvant agents to reduce the dose of first-line drugs and to improve first-line efficacy in refractory CIDP. It has been shown that mycophenolate mofetil tablets have fewer adverse effects than other immunosuppressive drugs such as cyclophosphamide and cyclosporin (3). The patient’s condition was stabilized after the fourth hospitalization by the use of mycophenolate as an adjuvant to reduce the therapeutic dose of glucocorticoids, taking into account the aggravation and recurrence of symptoms during the gradual reduction of glucocorticoids after the first and second discharge, as well as the side effects of glucocorticoid treatment, such as moon face, increased blood glucose, and decreased blood calcium. In this case, after the therapy of repeat IVIg was chosen, the clinical symptoms and EDX improved and it appeared that her disease fluctuations were effectively controlled. We observed a complete reversal in the ganglioside autoantibody spectrum results after multiple rounds of IVIg and corticosteroid treatments. She initially responded well to corticosteroids but then felt a relapse of symptoms when the dose decreased. After discharge, the patient experienced acute and recurrent episodes of symptoms after suffering from a flu infection. It is not clear whether the symptoms were caused by dose reduction or infection, but there was no manifestation of cranial nerve involvement, which we speculated might be the effect of the drug. Several randomized controlled trials have demonstrated that IVIg leads to improvement in most patients within 6 weeks of treatment, while the time to improve with corticosteroids usually takes several months. The patient needed long-term maintenance treatment.

This case contributes to the awareness that if initial treatment for CIDP fails or if the disease follows a relapse-remission pattern, it is necessary to consider misdiagnosis, other immune disorders, and insufficient immunotherapy doses. Furthermore, it is believed that antiganglioside and anti-sulfatide antibodies are closely related to CIDP pathogenesis and can assist in the diagnosis of the disease.

## Data availability statement

The original contributions presented in the study are included in the article/supplementary material. Further inquiries can be directed to the corresponding author.

## Ethics statement

Written informed consent was obtained from the individual(s) for the publication of any potentially identifiable images or data included in this article.

## Author contributions

RS: Writing – original draft, Writing – review & editing. YM: Investigation, Writing – review & editing. LL: Investigation, Writing – review & editing. W-HC: Investigation, Writing – review & editing. JX: Writing – review & editing. PL: Writing – review & editing. YD: Writing – review & editing.

## References

[1] Cabasson S Tardieu M Meunier A Rouanet-Larriviere MF Boulay C Pedespan JM. Childhood CIDP: Study of 31 patients and comparison between slow and rapid-onset groups. Brain Dev. (2015) 37:943–51. doi: 10.1016/j.braindev.2015.04.001 25921353

[2] Ruts L van Koningsveld R van Doorn PA. Distinguishing acute-onset CIDP from Guillain-Barré syndrome with treatment related fluctuations. Neurology. (2005) 65:138–40. doi: 10.1212/01.wnl.0000167549.09664.b8 16009902

[3] Van den Bergh PYK van Doorn PA Hadden RDM Avau B Vankrunkelsven P Allen JA. European Academy of Neurology/Peripheral Nerve Society guideline on diagnosis and treatment of chronic inflammatory demyelinating polyradiculoneuropathy: Report of a joint Task Force-Second revision. Eur J Neurol. (2021) 28:3556–83. doi: 10.1111/ene.14959 34327760

[4] Filosto M Cotelli M Todeschini A Broglio L Vielmi V Rinaldi F. Clinical spectrum and evolution of monoclonal gammopathy-associated neuropathy: an observational study. neurol. (2012) 18:378–84. doi: 10.1097/NRL.0b013e31826a99e9 23114670

[5] Tan CY Yahya MA Goh KJ Shahrizaila N. Nerve ultrasound score in chronic inflammatory demyelinating polyneuropathy. Medicina (Kaunas Lithuania). (2023) 59:747. doi: 10.3390/medicina59040747 37109705 PMC10144993

[6] Grimm A Oertl H Auffenberg E Schubert V Ruschil C Axer H. Differentiation between Guillain-Barré Syndrome and acute-onset chronic inflammatory demyelinating polyradiculoneuritis-a prospective follow-up study using ultrasound and neurophysiological measurements. Neurotherapeutics. (2019) 16:838–47. doi: 10.1007/s13311-019-00716-5 PMC6694337 30756363

[7] Preisner F Pitarokoili K Lueling B Motte J Fisse AL Grüter T. Quantitative magnetic resonance neurography in chronic inflammatory demyelinating polyradiculoneuropathy: A longitudinal study over 6 years. Ann Clin Trans Neurol. (2024) 11:593–606. doi: 10.1002/acn3.51978 PMC10963304 38111964

[8] Niu J Zhang L Fan J Liu J Ding Q Guan Y. Nerve ultrasound may help predicting response to immune treatment in chronic inflammatory demyelinating polyradiculoneuropathy. Neurol Sci. (2022) 43:3929–37. doi: 10.1007/s10072-022-05882-7 35061135

[9] Ruts L Drenthen J Jacobs BC van Doorn PA Dutch GBS Study Group. Distinguishing acute-onset CIDP from fluctuating Guillain-Barre syndrome: a prospective study. Neurology. (2010) 74:1680–6. doi: 10.1212/WNL.0b013e3181e07d14 20427754

[10] Ritter C Bobylev I Lehmann HC. Chronic inflammatory demyelinating polyneuropathy (CIDP): change of serum IgG dimer levels during treatment with intravenous immunoglobulins. J Neuroinflamm. (2015) 12:148. doi: 10.1186/s12974-015-0361-1 PMC4535537 26268846

[11] Querol L Siles AM Alba-Rovira R Jáuregui A Devaux J Faivre-Sarrailh C. Antibodies against peripheral nerve antigens in chronic inflammatory demyelinating polyradiculoneuropathy. Sci Rep. (2017) 7:14411. doi: 10.1038/s41598-017-14853-4 29089585 PMC5663697

[12] Kannan MA Ch RK Jabeen SA Mridula KR Rao P Borgohain R. Clinical, electrophysiological subtypes and antiganglioside antibodies in childhood Guillain-Barré syndrome. Neurol India. (2011) 59:727–32. doi: 10.4103/0028-3886.86549 22019659

[13] Fan C Jin H Hao H Gao F Sun Y Lu Y. Anti-ganglioside antibodies in Guillain-Barré syndrome and chronic inflammatory demyelinating polyneuropathy in Chinese patients. Muscle Nerve. (2017) 55:470–5. doi: 10.1002/mus.25266 27464289

[14] Cutillo G Saariaho AH Meri S. Physiology of gangliosides and the role of antiganglioside antibodies in human diseases. Cell Mol Immunol. (2020) 17:313–22. doi: 10.1038/s41423-020-0388-9 PMC7109116 32152553

[15] Chen L Zhang Y Qin N Han R Li Y. Atypical chronic inflammatory demyelinating polyradiculoneuropathy with ophthalmoplegia and anti-sulfatide IgM positivity. J Int Med Res. (2023) 51:3000605231193575. doi: 10.1177/03000605231193575 37812511 PMC10563483

[16] Pestronk A Li F Griffin J Feldman EL Cornblath D Trotter J. Polyneuropathy syndromes associated with serum antibodies to sulfatide and myelin-associated glycoprotein. Neurology. (1991) 41:357–62. doi: 10.1212/WNL.41.3.357 1706491

[17] Bus SRM Broers MC Lucke IM Bunschoten C van Lieverloo GGA Adrichem ME. Clinical outcome of CIDP one year after start of treatment: a prospective cohort study. J Neurol. (2022) 269:945–55. doi: 10.1007/s00415-021-10677-5 PMC8782785 34173873

[18] van Schaik IN Eftimov F van Doorn PA Brusse E van den Berg LH van der Pol WL. Pulsed high-dose dexamethasone versus standard prednisolone treatment for chronic inflammatory demyelinating polyradiculoneuropathy (PREDICT study): a double-blind, randomised, controlled trial. Lancet Neurol. (2010) 9:245–53. doi: 10.1016/S1474-4422(10)70021-1 20133204

